# Placental Mesenchymal Stem Cells Alleviate Podocyte Injury in Diabetic Kidney Disease by Modulating Mitophagy via the SIRT1-PGC-1alpha-TFAM Pathway

**DOI:** 10.3390/ijms24054696

**Published:** 2023-02-28

**Authors:** Xiudan Han, Jiao Wang, Ruilin Li, Meiling Huang, Guanru Yue, Lulu Guan, Yuanyuan Deng, Wei Cai, Jixiong Xu

**Affiliations:** 1 Department of Endocrinology and Metabolism, First Affiliated Hospital of Nanchang University, Nanchang 330006, China; 2 Jiangxi Clinical Research Center for Endocrine and Metabolic Disease, Nanchang 330006, China; 3 Jiangxi Branch of National Clinical Research Center for Metabolic Disease, Nanchang 330006, China; 4 Department of Cell Biology, School of Medicine, Nanchang University, Nanchang 330006, China

**Keywords:** diabetic kidney disease, placenta derived mesenchymal stem cells, podocyte injury, mitophagy, SIRT1-PGC-1α-TFAM pathway

## Abstract

The use of mesenchymal stem cells (MSCs) has become a new strategy for treating diabetic kidney disease (DKD). However, the role of placenta derived mesenchymal stem cells (P-MSCs) in DKD remains unclear. This study aims to investigate the therapeutic application and molecular mechanism of P-MSCs on DKD from the perspective of podocyte injury and PINK1/Parkin-mediated mitophagy at the animal, cellular, and molecular levels. Western blotting, reverse transcription polymerase chain reaction, immunofluorescence, and immunohistochemistry were used to detect the expression of podocyte injury-related markers and mitophagy-related markers, SIRT1, PGC-1α, and TFAM. Knockdown, overexpression, and rescue experiments were performed to verify the underlying mechanism of P-MSCs in DKD. Mitochondrial function was detected by flow cytometry. The structure of autophagosomes and mitochondria were observed by electron microscopy. Furthermore, we constructed a streptozotocin-induced DKD rat model and injected P-MSCs into DKD rats. Results showed that as compared with the control group, exposing podocytes to high-glucose conditions aggravated podocyte injury, represented by a decreased expression of Podocin along with increased expression of Desmin, and inhibited PINK1/Parkin-mediated mitophagy, manifested as a decreased expression of Beclin1, the LC3II/LC3I ratio, Parkin, and PINK1 associated with an increased expression of P62. Importantly, these indicators were reversed by P-MSCs. In addition, P-MSCs protected the structure and function of autophagosomes and mitochondria. P-MSCs increased mitochondrial membrane potential and ATP content and decreased the accumulation of reactive oxygen species. Mechanistically, P-MSCs alleviated podocyte injury and mitophagy inhibition by enhancing the expression of the SIRT1-PGC-1α-TFAM pathway. Finally, we injected P-MSCs into streptozotocin-induced DKD rats. The results revealed that the application of P-MSCs largely reversed the markers related to podocyte injury and mitophagy and significantly increased the expression of SIRT1, PGC-1α, and TFAM compared with the DKD group. In conclusion, P-MSCs ameliorated podocyte injury and PINK1/Parkin-mediated mitophagy inhibition in DKD by activating the SIRT1-PGC-1α-TFAM pathway.

## 1. Introduction

Diabetic kidney disease (DKD), also known as diabetic nephropathy, is a renal complication of diabetes, is one of the main causes of morbidity and mortality in patients with diabetes mellitus [1]. Epidemiological studies have shown that, compared with other diabetic complications, the prevalence of DKD has not changed significantly in recent decades [2]. DKD has become a global public health issue and has imposed a tremendous economic burden onto society and public health systems. Nearly 40% of people with diabetes mellitus will develop DKD, which is the leading cause of chronic kidney disease and end-stage renal disease worldwide [3]. Albuminuria is a significant clinical symptom of DKD and is closely associated with podocyte damage [4]. Podocytes are terminally differentiated glomerular visceral epithelial cells that can maintain the integrity of the glomerular filtration barrier [5,6]. The injury and loss of podocytes comprise an early feature of DKD that predicts its progressive course [7]. Silencing information regulator 2 related enzyme 1 (sirtuin1, SIRT1), an NAD(+)-regulated deacetylase, plays a significant role in cellular senescence [8]. Protective effects of SIRT1 on podocyte injury in DKD have been reported [9,10]. Hence, maintaining the normal structure and function of podocytes and preventing podocyte damage are important measures in the prevention and treatment of DKD.

Autophagy plays a crucial role in maintaining lysosome homeostasis in podocytes under diabetic conditions, and its impairment is an important pathophysiological mechanism of DKD [11]. Mitophagy is a highly conserved autophagic process that selectively removes damaged or unnecessary mitochondria, and it plays an important role in maintaining the stability of the intracellular environment [12]. Phosphatase and tensin homolog-induced kinase 1 (PINK1)/Parkin-mediated mitophagy is a hotspot for research in mammalian cells [13]. Damaged mitochondria accumulate PINK1 in the mitochondrial outer membrane, which then recruits and activates Parkin, resulting in the ubiquitination of mitochondrial proteins. These proteins can then be bound by the autophagic proteins p62/SQSTM1 and LC3, resulting in the degradation of mitochondria by mitophagy [14]. Peroxisome proliferator-activated receptor γ coactivator-1alpha (PGC-1alpha, PGC-1α) and transcription factor A, mitochondrial (TFAM) are involved in mitochondrial biogenesis [15]. Impaired mitophagy and persistent mitochondrial dysfunction play a crucial role in the early stages and progression of DKD [16,17]. Unfortunately, there is no efficient therapy to prevent or even reverse podocyte injury and mitophagy inhibition in DKD.

In recent years, the application of mesenchymal stem cells (MSCs) in the treatment of DKD has shown good prospects [18,19]. Placenta derived mesenchymal stem cells (P-MSCs) have many advantages, such as an extensive sources, convenient drawing, less ethical controversy, and strong proliferative ability. Studies have shown that human umbilical cord MSCs prevented podocyte damage in DKD by inhibiting the Toll-like receptor signaling pathway and depressing inflammation [20]. However, no studies have reported the effect of P-MSCs on podocyte injury and mitophagy in DKD, and the underlying mechanism remains unclear. From this background, we will investigate the therapeutic effect of P-MSCs on DKD and the corresponding molecular mechanism from the perspective of podocyte injury and mitophagy inhibition.

## 2. Results

### 2.1. Podocyte Injury and PINK1/Parkin-Mediated Mitophagy Inhibition Induced by High Glucose in the Mouse Podocyte Cell Line

**Figure 1:**
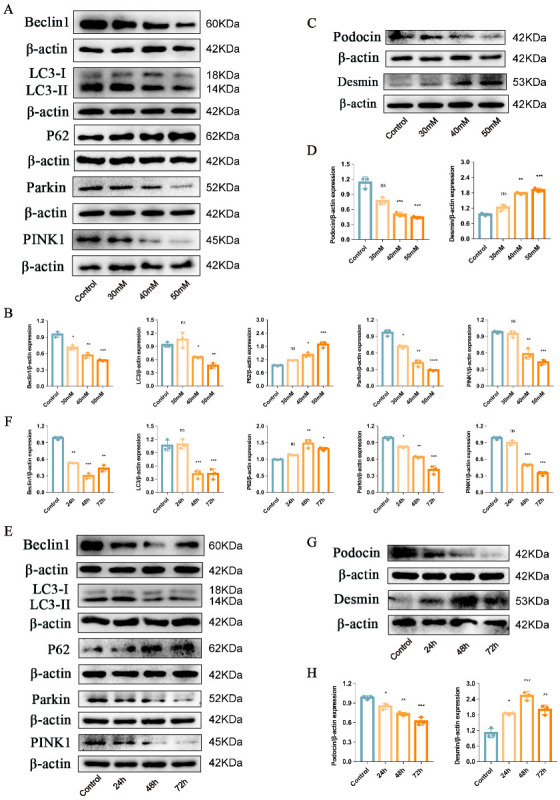
Podocyte injury and PINK1/Parkin-mediated mitophagy inhibition induced by HG. (**A**,**B**) Representative Western blot analysis of Beclin1, LC3II/LC3I ratio, P62, Parkin, and PINK1 in MPC5 treated with different concentrations of glucose. (**C**,**D**) Representative Western blot analysis of Podocin and Desmin in MPC5 treated with different concentrations of glucose. (**E**,**F**) Representative Western blot analysis of Beclin1, LC3II/LC3I ratio, P62, Parkin, and PINK1 in MPC5 treated with HG for 24 h, 48 h, and 72 h. (**G**,**H**) Representative Western blot analysis of Podocin and Desmin in MPC5 treated with HG for 24 h, 48 h, and 72 h. *n* = 3 for (**A**–**H**). β-actin was used as loading control. * *p* < 0.05, ** *p* < 0.01, *** *p* < 0.001, and **** *p* < 0.0001 vs. control; ns = not significant.

First, we determined whether high glucose (HG)-aggravated podocyte injury and decreased PINK1/Parkin-mediated mitophagy. We selected the mouse podocyte cell line, the MPC5 cell line, for the cell experiments. MPC5 was treated with different concentrations of glucose (30 mM, 40 mM, and 50 mM) for 48 h. The results showed that as compared with the control group, exposing podocytes to HG conditions inhibited PINK1/Parkin-mediated mitophagy, manifested as a decreased expression of Beclin1, the LC3II/LC3I ratio, Parkin, PINK1 associated with an increased expression of P62 (Figure 1A,B), and aggravated podocyte injury, represented by a decreased expression of Podocin along with an increased expression of Desmin (Figure 1C,D). Moreover, HG suppressed PINK1/Parkin-mediated mitophagy and exacerbated podocyte injury in a concentration-dependent manner. Then, to evaluate the optimal intervention time for HG, we treated MPC5 with HG (50 mM) for 24, 48, and 72 h. Results suggested that the expression of Beclin1 and the LC3II/LC3I ratio were significantly decreased, and the expression of P62 and Desmin were significantly increased after 48 h of HG intervention compared with 24 h and 72 h (Figure 1E–H). Hence, in the subsequent experiments, HG was used at 50 mM for 48 h.

### 2.2. P-MSCs Attenuated HG-Induced Podocyte Injury and PINK1/Parkin-Mediated Mitophagy Inhibition

**Figure 2:**
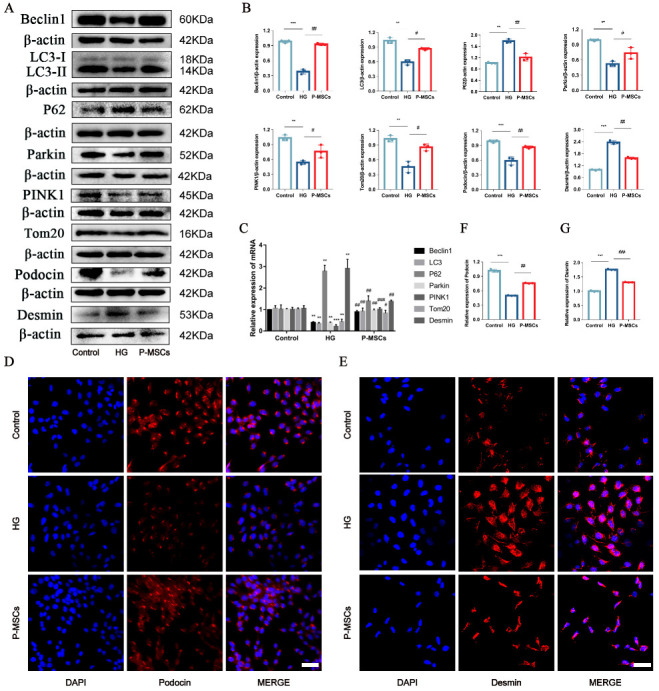
P-MSCs attenuated HG-induced podocyte injury and PINK1/Parkin-mediated mitophagy inhibition. (**A**,**B**) Representative Western blot analysis of Beclin1, LC3II/LC3I ratio, P62, Parkin, PINK1, and Tom20 as well as Podocin and Desmin in MPC5. (**C**) Representative RT-PCR analysis of Beclin1, LC3II/LC3I ratio, P62, Parkin, PINK1, and Tom20, as well as Desmin in MPC5. (**D**–**G**) Immunofluorescence staining of Podocin and Desmin in MPC5 (Magnification, 400×). Scale bars: 50 μm for (**D**,**E**). *n* = 3 for (**A**–**E**). Values are expressed as the mean ± SD. β-actin was used as loading control. ** *p* < 0.01 and *** *p* < 0.001 vs. control, ^#^ *p* < 0.05, ^##^ *p* < 0.01, and ^###^ *p* < 0.01 vs. HG.

In recent years, MSCs have played an increasingly crucial role in diabetes and its complications [21,22]. We investigated whether P-MSCs could ameliorate podocyte injury and regulate PINK1/Parkin-mediated mitophagy in DKD. To this purpose, we used western blot (WB) and reverse transcription–polymerase chain reaction (RT-PCR) to detect PINK1/Parkin-mediated mitophagy/podocyte injury-related proteins and mRNAs in MPC5 cells. WB analysis showed that the P-MSCs group had an increased expression of Beclin1, the LC3II/LC3I ratio, Parkin, PINK1, Tom20, and Podocin as compared with the HG group, and a decreased expression of P62 and Desmin (Figure 2A,B). The results of the RT-PCR analysis were consistent with those of the WB analysis, except for Podocin (Figure 2C). Furthermore, immunofluorescence analysis revealed that P-MSCs attenuated podocyte injury by increasing the expression of Podocin and decreasing the expression of Desmin (Figure 2D–G). These results illustrated that P-MSCs could reduce the degree of HG-induced podocyte injury and increase the level of PINK1/Parkin-mediated mitophagy.

**Figure 3:**
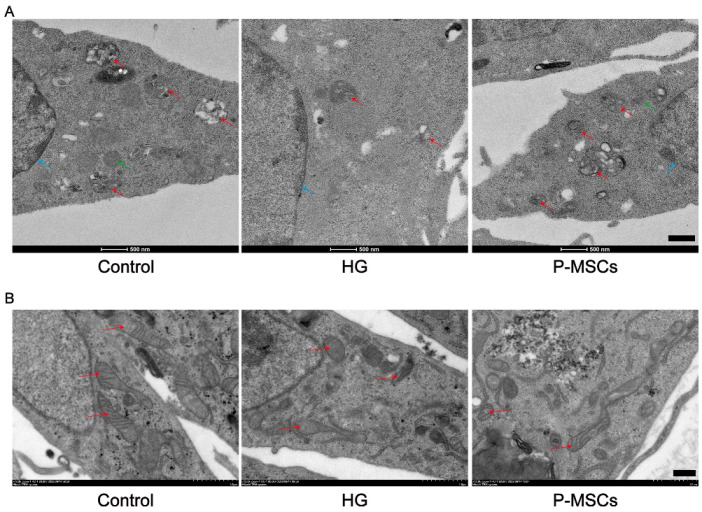
Transmission electron microscopy analysis of autophagosomes and mitochondria. (**A**) The integrity of the nuclear membrane was disrupted (blue arrows) accompanied by fewer autophagosomes (red arrows) and lysosomes (green arrows) in the HG group compared with the control group. P-MSCs reduced nuclear damage and increased autophagosomes and lysosomes. (**B**) Mitochondria were columnar or reticular, with clear mitochondrial cristae, normal matrix density, and intact mitochondrial membranes in the control group. Mitochondrial structures were significantly damaged in the HG group. P-MSCs improved the structures of mitochondria. Scale bars: 500 nm for (**A**,**B**).

In addition, we used transmission electron microscopy to observe the structure and quantity of the nuclear membrane, autophagosomes, and lysosomes. As shown in Figure 3A, the nuclear membrane was intact along with more autophagosomes (red arrows) and lysosomes (green arrows) in the control group. However, the integrity of the nuclear membrane was disrupted, and was accompanied by fewer autophagosomes and lysosomes under HG conditions. Interestingly, P-MSCs reduced nuclear damage and increased autophagosomes and lysosomes.

### 2.3. P-MSCs Extenuated HG-Mediated Mitochondrial Dysfunction and Reactive Oxygen Species Accumulation

**Figure 4:**
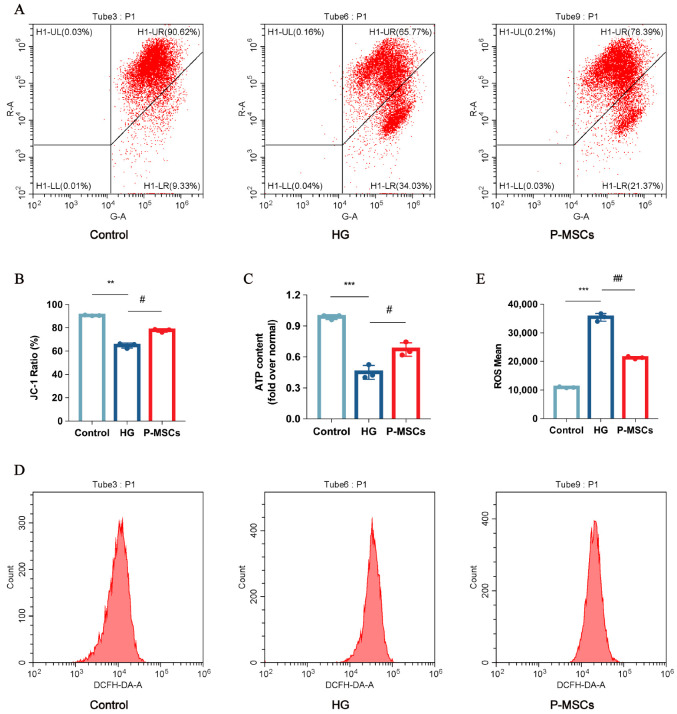
P-MSCs extenuated HG-mediated mitochondrial dysfunction and ROS accumulation. (**A**) Mitochondrial membrane potential was measured by flow cytometry. (**B**) Mitochondrial membrane potential was quantified in MPC5. (**C**) ATP levels were quantified in MPC5. (**D**) ROS was measured by flow cytometry. (**E**) ROS were quantified in MPC5. *n* = 3 for (**A**–**E**). Data are shown as the means ± SD from three independent experiments. ** *p* < 0.01 and *** *p* < 0.001 vs. control, ^#^ *p* < 0.05 and ^##^ *p* < 0.01 vs. HG.

We observed mitochondria structure using transmission electron microscopy. As shown in Figure 3B, mitochondria were columnar or reticular, with clear mitochondrial cristae, normal matrix density, and intact mitochondrial membranes in the control group. Mitochondrial structures were significantly damaged in the HG group. Nevertheless, P-MSCs improved the structure of mitochondria (Figure 3B). Additionally, a growing number of studies have implicated that mitochondrial dysfunction was associated with podocyte damage and albuminuria [23,24]. In this study, we measured mitochondrial membrane potential (ΔΨm) and ATP content in different groups, representing the level of mitochondrial function. The results implied that ΔΨm (Figure 4A,C) and ATP content (Figure 4D) were significantly decreased in the HG group as compared with the control group, indicating that mitochondrial function was seriously damaged under HG states. Furthermore, in conditions of hyperglycemia, accumulation of reactive oxygen species (ROS) was observed (Figure 4B,E), which indirectly contributes to podocyte injury. Interestingly, the effects of HG on ΔΨm, ROS, and ATP content were reversed by P-MSCs, suggesting an overall improvement of mitochondrial function (Figure 4A–E).

### 2.4. P-MSCs Alleviated HG-Induced Podocyte Injury and PINK1/Parkin-Mediated Mitophagy Inhibition by Activating the SIRT1-PGC-1α-TFAM Signaling Pathway

**Figure 5:**
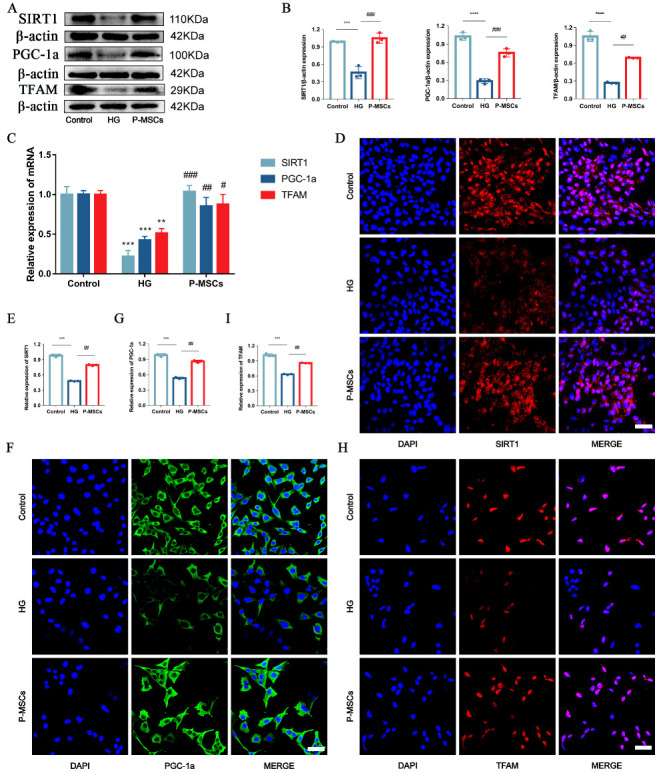
P-MSCs increased the expressions of SIRT1, PGC-1a, and TFAM in MPC5. (**A**,**B**) Representative Western blot analysis of SIRT1, PGC-1a, and TFAM in MPC5. (**C**) Representative RT-PCR analysis of SIRT1, PGC-1a, and TFAM in MPC5. D-I: Immunofluorescence staining of SIRT1, PGC-1a, and TFAM in MPC5 (Magnification, 400×). Scale bars: 50 μm for (**D**,**F**,**H**). *n* = 3 for (**A**–**I**). Values are expressed as the mean ± SD. Β-actin was used as loading control. ** *p* < 0.01, *** *p* < 0.001, and **** *p* < 0.0001 vs. control, ^#^ *p* < 0.05, ^##^ *p* < 0.01, and ^###^ *p* < 0.001 vs. HG.

We performed the following experiments to further explore the mechanism by which P-MSCs affected podocyte injury and PINK1/Parkin-mediated mitophagy. Recent studies have reported that mitophagy is initiated by upregulation of SIRT1 [25,26]. In addition, PGC-1α and TFAM proteins appear to be vital factors in SIRT1-associated mitophagy [27,28]. Hence, we detected the expression levels of SIRT1, PGC-1α, and TFAM in different groups first and foremost. The results of the WB and RT-PCR analyses suggested that the expressions of SIRT1, PGC-1α, and TFAM were notably decreased in the HG group as compared with the control group. However, the influence of HG on the expressions of SIRT1, PGC-1α, and TFAM was reverted by P-MSCs (Figure 5A–C). Immunofluorescence analysis also consistently revealed the same results as WB and RT-PCR analyses (Figure 5D–I).

**Figure 6:**
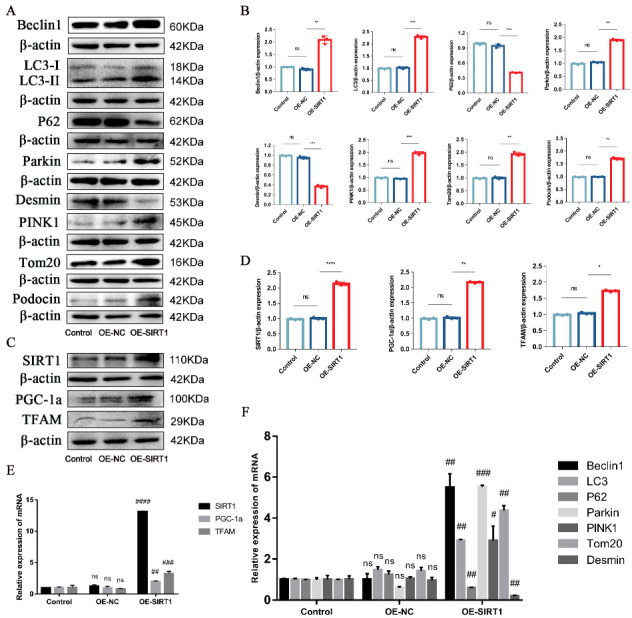
SIRT1 overexpression alleviated podocyte injury and PINK1/Parkin-mediated mitophagy inhibition. (**A**,**B**) Representative Western blot analysis of Beclin1, LC3II/LC3I ratio, P62, Parkin, PINK1, and Tom20, as well as Podocin and Desmin in MPC5. (**C**,**D**) Representative Western blot analysis of SIRT1, PGC-1a, and TFAM in MPC5. (**E**) Representative RT-PCR analysis of SIRT1, PGC-1a, and TFAM in MPC5. (**F**) Representative RT-PCR analysis of Beclin1, LC3II/LC3I ratio, P62, Parkin, PINK1, and Tom20, as well as Desmin in MPC5. *n* = 3 for (**A**–**F**). β-actin was used as loading control. * *p* < 0.05, ** *p* < 0.01, *** *p* < 0.001 and **** *p* < 0.0001 vs. the negative control vector (OE-NC). ^#^ *p* < 0.05, ^##^ *p* < 0.01, ^###^ *p* < 0.001, and ^####^ *p* < 0.0001 vs. OE-NC; ns = not significant.

Subsequently, to confirm whether SIRT1 was necessary for P-MSCs to regulate podocyte injury and PINK1/Parkin-mediated mitophagy, SIRT1 expression was overexpressed. We verified the overexpression efficiency using both WB analysis (Figure 6C,D) and RT-PCR analysis (Figure 6E). The changes of the podocyte injury and mitophagy-related proteins and mRNAs after SIRT1 overexpression were then detected again. WB analysis (Figure 6A,B) and RT-PCR analysis (Figure 6F) showed that as compared with the negative control vector (OE-NC) group, the expressions of Beclin1, the LC3II/LC3I ratio, Parkin, PINK1, Tom20, and Podocin were increased. In contrast, the expressions of P62 and Desmin were decreased in the SIRT1 overexpression (OE-SIRT1) group, indicating that by upregulating the expression of SIRT1, P-MSCs play a protective role in HG-induced podocyte injury and PINK1/Parkin-mediated mitophagy. Interestingly, we found that the expression of PGC-1α and TFAM was increased along with SIRT1 overexpression (Figure 6C–E). Furthermore, siRNA was used to knock down the expression of SIRT1. The WB and RT-PCR analyses determined podocyte injury-related markers and mitophagy-related markers. The results demonstrated that the inhibition of SIRT1 expression aggravated podocyte injury, inhibited PINK1/Parkin-mediated mitophagy, and decreased the expression of PGC-1α and TFAM (Figure S1A–G).

**Figure 7:**
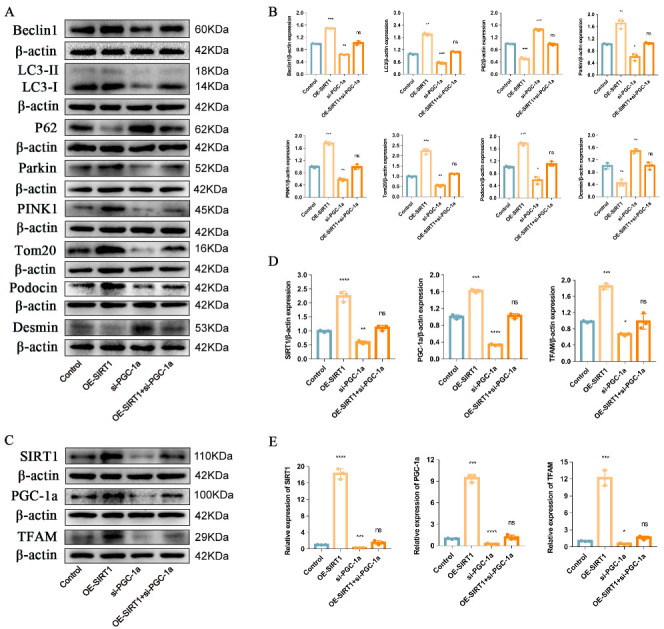
Overexpression of SIRT1 and inhibition of PGC-1a expression were performed (rescue experiments). (**A**,**B**) Representative Western blot analysis of Beclin1, LC3II/LC3I ratio, P62, Parkin, PINK1, and Tom20, as well as Podocin and Desmin in MPC5. (**C**,**D**) Representative Western blot analysis of SIRT1, PGC-1a, and TFAM in MPC5. (**E**) Representative RT-PCR analysis of SIRT1, PGC-1a, and TFAM in MPC5. *n* = 3 for (**A**–**E**). β-actin was used as loading control. * *p* < 0.05, ** *p* < 0.01, *** *p* < 0.001, and **** *p* < 0.0001 vs. control; ns = not significant.

In addition, to determine whether P-MSCs protected podocytes from damage and enhanced PINK1/Parkin-mediated mitophagy through the SIRT1-PGC-1α-TFAM signaling pathway, we performed rescue experiments. WB analysis data showed that the expressions of Beclin1, the LC3II/LC3I ratio, Parkin, PINK1, Tom20, and Podocin increased, whereas the expressions of P62 and Desmin decreased after SIRT1 overexpression. Nevertheless, when the expression of PGC-1α was inhibited, the expression of Beclin1, the LC3II/LC3I ratio, Parkin, PINK1, Tom20, and Podocin were decreased, and the expression of P62 and Desmin were increased. Notably, mitophagy-related proteins and podocyte injury-related proteins were reversed when SIRT1 was overexpressed and PGC-1α was inhibited at the same time, suggesting that P-MSCs play a protective role in podocyte injury and PINK1/Parkin-mediated mitophagy inhibition through the SIRT1-PGC-1α signaling pathway (Figure 7A,B). Furthermore, when the expression of SIRT1 was overexpressed, the expression of TFAM also increased correspondingly. Meanwhile, the expression of TFAM decreased along with the decrease of PGC-1α expression. Interestingly, when SIRT1 was overexpressed and PGC-1α was inhibited simultaneously, the expression of TFAM was reverted at both protein (Figure 7C,D) and mRNA (Figure 7E) levels, indicating that P-MSCs play a protective role in podocytes via the SIRT1-PGC-1α-TFAM signaling pathway.

### 2.5. P-MSCs Ameliorated Streptozotocin-Induced Podocyte Injury and PINK1/Parkin-Mediated Mitophagy in DKD Rats

**Figure 8:**
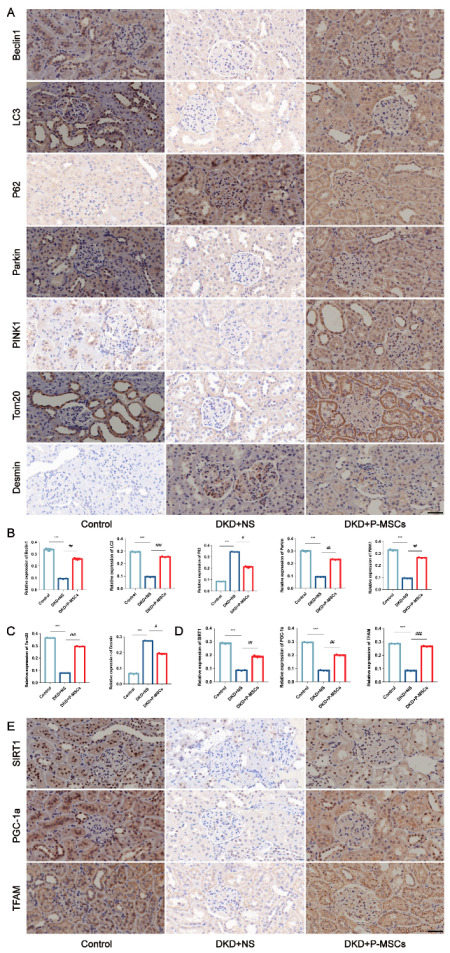
P-MSCs ameliorated STZ-induced podocyte injury and PINK1/Parkin-mediated mitophagy in DKD rats. (**A**–**C**) Immunohistochemical analysis of Beclin1, LC3, P62, Parkin, PINK1, and Tom20, as well as Desmin in DKD rats (Magnification, 400×). (**D**,**E**) Immunohistochemical analysis of SIRT1, PGC-1a, and TFAM in DKD rats (Magnification, 400×). Scale bars: 40 μm for (**A**,**E**). *n* = 6 rats/group for (**A**,**E**). Values are expressed as the mean ± SD. *** *p* < 0.001 vs. control, ^#^ *p* < 0.05, ^##^ *p* < 0.01, and ^###^ *p* < 0.001 vs. DKD + NS.

Our group had previously constructed an streptozotocin (STZ)-induced DKD rat model and successfully injected P-MSCs into DKD rats via the tail vein. The results showed that treatment with P-MSCs can effectively improve blood glucose, serum creatinine, blood urea nitrogen, urinary albumin/creatinine ratio, renal hypertrophy index, and renal pathological injury in DKD rats [29]. To validate the protective effect of P-MSCs on DKD rats in vivo from the perspective of podocyte injury and PINK1/Parkin-mediated mitophagy, we performed an immunohistochemical analysis in this study. The results showed that compared with the control group, the expression of Beclin1, LC3, Parkin, PINK1, and Tom20 were decreased, and the expression of P62 and Desmin were increased in the DKD group. However, the application of P-MSCs largely reversed the markers related to podocyte injury and PINK1/Parkin-mediated mitophagy (Figure 8A–C), meaning that P-MSCs could alleviate podocyte injury and PINK1/Parkin-mediated mitophagy inhibition in DKD rats. Furthermore, the expressions of SIRT1, PGC-1α, and TFAM were markedly decreased in the DKD group compared with the control group. Nevertheless, the injection of P-MSCs into DKD rats significantly increased the expression of SIRT1, PGC-1α, and TFAM in DKD rats (Figure 8D,E).

## 3. Discussion

The therapeutic effect of P-MSCs on DKD has not been reported until now. We evaluated for the first time whether P-MSCs ameliorated podocyte injury and PINK1/Parkin-mediated mitophagy inhibition in DKD and further explored the underlying molecular mechanisms. Based on our data, we can draw the following conclusions. First, we found that hyperglycemia induced podocyte injury and PINK1/Parkin-mediated mitophagy inhibition in the cell experiments. Second, P-MSCs not only alleviated HG-induced podocyte injury and PINK1/Parkin-mediated mitophagy inhibition but also prevented mitochondrial dysfunction. In addition, through knockdown, overexpression, and rescue experiments, we demonstrated that P-MSCs extenuated HG-induced podocyte injury and PINK1/Parkin-mediated mitophagy inhibition by activating the SIRT1-PGC-1α-TFAM signaling pathway. Finally, we further verified that P-MSCs improved renal function and attenuated podocyte injury and PINK1/Parkin-mediated mitophagy inhibition induced by STZ in DKD rats. Briefly, P-MSCs ameliorated podocyte injury and PINK1/Parkin-mediated mitophagy inhibition in DKD through the SIRT1-PGC-1α-TFAM signaling pathway. Targeting the PINK1/Parkin-mediated mitophagy and SIRT1-PGC-1α-TFAM signaling pathways may provide a new potential therapeutic approach for P-MSCs in DKD.

It is well known that podocytes are highly differentiated epithelial cells attached to the glomerular basement membrane and play a significant role in maintaining the normal filtration function of the kidney [30]. Podocyte injury is a crucial factor in DKD progression [31]. Hence, we wanted to know whether P-MSCs improved podocyte injury in DKD. Previous validation studies have shown that Desmin, a podocyte injury marker, was upregulated in DKD. In contrast, Podocin, a key component of the podocyte slit diaphragm, was downregulated [32,33,34]. These results were consistent with our findings. However, the effect of P-MSCs on podocyte injury in DKD was investigated in this study. Our results found that P-MSCs increased the expression of Podocin and decreased the expression of Desmin, implying that P-MSCs could indeed alleviate podocyte injury in DKD.

Mitophagy is a process in which damaged or dysfunctional mitochondria are selectively delivered to lysosomes for degradation [35]. In mammals, it is primarily regulated by the PINK1/Parkin signaling pathway. PINK1/Parkin-mediated mitophagy contributes to maintaining mitochondrial quantity and quality in a variety of cell types [13]. Recent observations have reported that PINK1/Parkin-mediated mitophagy is one pathogenesis of DKD [36,37]. P62/SQSTM1, as an autophagy adaptor, interacts with LC3 and then participates in the process of PINK1/Parkin-mediated mitophagy [38]. Tom20, a functional protein of mitochondria, was associated with mitophagy and mitochondrial function [39]. He et al. showed that when the level of mitophagy was reduced, Beclin1, the LC3II/LC3I ratio, Parkin, PINK1, and Tom20 levels increased and P62 levels decreased. Our results are in accordance with previous studies.

We next evaluated the effect of P-MSCs on PINK1/Parkin-mediated mitophagy in DKD. A growing but limited number of studies have found that MSCs can improve cell metabolism and function through mitophagy [40,41] and MSCs prevent the progression of DKD by reversing mitochondrial dysfunction in renal tubular epithelial cells [42]. P-MSCs have the advantages of abundant sources, strong proliferation potential, and low immunogenicity, which make them a valuable biological resource for the promotion of tissue repair. A previous study reported that P-MSCs can improve tissue damage of the testis by promoting autophagy and reducing apoptosis [43]. Li et al. demonstrated that P-MSCs can reduce the damage of pulmonary microvascular endothelial cells and improve mitochondrial function by enhancing autophagy [44]. Some studies have also shown that P-MSCs upregulated markers related to mitophagy and adjusted mitochondrial energy metabolism in trophoblast cells [45,46]. However, no studies have reported the ability of P-MSCs to repair renal injury through mitophagy in DKD. In this study, we found that P-MSCs increased the levels of mitophagy-related markers in in vitro and in vivo experiments. The results of WB, RT-PCR, and immunohistochemical analysis showed that as compared with the HG group, the P-MSCs group increased the expression of Beclin1, the LC3II/LC3I ratio, Parkin, and PINK1 and decreased the expression of P62. Moreover, P-MSCs reduced ROS accumulation and mitochondrial dysfunction, which was manifested by the increase in ΔΨm and ATP content.

Finally, we further explored the mechanism of P-MSCs on podocyte injury and PINK1/Parkin-mediated mitophagy in DKD. SIRT1 has good potential as a clinical target for preventing and treating DKD [47]. By increasing the expression of SIRT1, MSCs can reduce inflammasome signaling and apoptosis [48]. Studies have also suggested that the expression of PGC-1α and TFAM are decreased in human podocytes under HG [49]. Mitochondria from MSCs were transferred to macrophages in a co-culture system consisting of MSCs and macrophages. MSCs also ameliorated kidney injury in mice with DKD through mitochondrial transfer, which is dependent on PGC-1α-mediated mitochondrial biogenesis [50]. TFAM is essential for maintaining mitochondrial DNA and mitochondrial biogenesis [51]. Furthermore, the activation of the SIRT1/PGC-1α pathway can increase the level of mitophagy [52], and the SIRT1-PGC-1α-TFAM pathway played a crucial role in regulating mitochondrial function [28]. Based on the above reported literature, we wanted to prove whether P-MSCs alleviated podocyte injury and PINK1/Parkin-mediated mitophagy through the SIRT1-PGC-1α-TFAM signaling pathway. The results of the WB, RT-PCR, immunofluorescence, and immunohistochemistry analyses confirmed that, as compared with the control group, the expression of SIRT1, PGC-1α, and TFAM in the HG group was significantly decreased. The effects of HG on SIRT1, PGC-1α, and TFAM were reversed by P-MSCs. Furthermore, we also demonstrated that P-MSCs attenuated podocyte injury and PINK1/Parkin-mediated mitophagy inhibition via the activation of the SIRT1-PGC-1α-TFAM signaling pathway through knockdown, overexpression, and rescue experiments. Our results suggest that the SIRT1-PGC-1α-TFAM signaling pathway plays an important role in the attenuation of podocyte injury and PINK1/Parkin-mediated mitophagy in DKD for P-MSCs.

We not only evaluated the therapeutic efficacy and cellular mechanisms of P-MSCs on DKD, but also affirmed that the therapeutic measures of P-MSCs are safe and effective. However, our study also has some limitations. Firstly, in our vivo experiments, we merely demonstrated the role of P-MSCs in STZ rats instead of db/db mice. However, rats are more similar to humans in cognitive behavior compared to mice. Secondly, in vitro experiments, we only investigated the beneficial effects of P-MSCs on podocyte injury and mitophagy in DKD, so the protective effects of P-MSCs on renal tubular cell injury require further evaluation. Finally, it has been reported in the literature that P-MSCs provide promising applications for clinical treatments [53]. However, we did not continue to further explore the mechanism of P-MSCs in DKD at the organizational level. Consequently, we will continue to carry out relevant clinical studies for the benefit of patients with DKD.

In summary, our findings provide important experimental evidence that P-MSCs play an essential role in the treatment of DKD, not only at the cellular level, but also at the animal level. We probed the therapeutic effect of P-MSCs in DKD mainly from the perspective of podocyte injury and PINK1/Parkin-mediated mitophagy inhibition. Interestingly, we detected that the SIRT1-PGC-1α-TFAM signaling pathway played a crucial role in DKD.

## 4. Materials and Methods

### 4.1. Reagents and Antibodies

Fetal bovine serum (FBS) was purchased from Gibco (HyClone, Logan, UT, USA). Lipofectamine 2000 was acquired from Invitrogen (Waltham, MA, USA). Anti-Beclin1 (11306-1-AP), anti-LC3B (14600-1-AP), anti-PINK1 (23274-1-AP), anti-Tom20 (11802-1-AP), anti-PGC-1α (66369-1-Ig), and anti-Podocin (20384-1-AP) were purchased from Proteintech (Rosemont, IL, USA). Anti-P62 (ab109012), anti-Parkin (ab77924), anti-Desmin (ab32362), and anti-SIRT1 (ab189494) were purchased from Abcam (Boston, MA, USA). TFAM (AF0531) was obtained from Affinity Biosciences (Cincinnati, OH, USA). Anti-beta actin (anti-β-actin) and horseradish peroxidase-conjugated secondary antibodies were acquired from Beijing Zhong Shan Golden Bridge Biological Technology Co., Ltd. (Beijing, China).

### 4.2. Cell Culture

The MPC5 cell line was purchased from GuangZhou Jennio Biotech Co., Ltd. (Guangzhou, China). P-MSCs were kindly provided and prepared in the GMP laboratory of the Stem Cell Engineering Research Center of Jiangxi Province (Shangrao, China). P-MSCs were isolated based on methods previously described [54,55,56]. Briefly, human placenta was obtained from a healthy mother. Informed consent was obtained from participants in all studies. Placental tissues were treated with collagenase II (Gibco, Grand Island, NY, USA) at 37 °C for 1 h and further digested with trypsin (Gibco) at 37 °C for 30 min with gentle agitation. The surface markers and differentiation capacity of P-MSCs have been previously identified [57].

P-MSCs and MPC5 were seeded separately in T25 flasks (Corning, NY, USA) and cultured in Dulbecco’s Modified Eagle’s Medium (Gibco) containing 10% FBS at 37 °C in a 5% CO_2_ humidified incubator. MPC5 was divided into different groups, which were treated with normal glucose (5.6 mM), HG (30 mM, 40 mM, and 50 mM), and HG plus P-MSCs. P-MSCs and MPC5 were co-cultured at a ratio of 1:10.

### 4.3. Transfections of Plasmids and Small Interfering RNAs

To effect changes in SIRT1, MPC5 was treated with SIRT1 plasmid using Lipofectamine 2000 as the transfection reagent and nonsense strand negative control (NC) as controls. Full sequences of SIRT1 plasmid can be found in Supplementary Table S1. Briefly, cells were starved for 2 h in six-well plates before transfection. Lipofectamine 2000 was mixed with 250 μL Opti-MEM, whereas SIRT1 plasmid was mixed with 250 μL Opti-MEM at a 1 μg target dose (the plasmid group was supplemented with 10 μL Lipofectamine 2000 reagent/well). The two commixtures were mixed together for 20 min and then added to the cell culture medium. After 6 to 8 h, the transfection medium was removed, and the cells were treated with corresponding stimuli. Cells were incubated for 48 h, and then collected for subsequent experiments.

Predesigned and validated small interfering RNAs (siRNAs) specific for SIRT1 (sense: CAUCUUGCCUGAUUUGUAATT; antisense: UUACAAAUCAGGCAAGAUGTT), PGC-1α (sense: CCAAGACUCUAGACAACUATT; antisense: UAGUUGUCUAGAGUCUUGGTT), and NC were obtained from Santa Cruz Biotechnology (Santa Cruz, CA, USA). Transfection was performed using Lipofectamine 2000 according to the manufacturer’s instructions. Transfection and intervention were similar to the experiments described in the previous phase. Each experiment was carried out at least in triplicate. 

### 4.4. WB Assay

Cell total protein was extracted using RIPA lysis buffer (APPLYGEN) supplemented with a protease inhibitor and phosphatase inhibitor (GLPBIO). The protein concentration was quantified by the bicinchoninic acid protein assay kit (TransGen Biotech, Beijing, China). Equal amounts of protein samples were separated by sodium dodecyl sulfate/polyacrylamide gel electrophoresis and then transferred to polyvinylidene difluoride membranes (Millipore, Burlington, MA, USA). After sealing with 5% nonfat dry milk in phosphate-buffered saline (PBS) with Tween 20 (PBST) for 60 min, the membrane was incubated with the primary antibodies anti-Beclin1 (1:1000), anti-Lc3B (1:2000), anti-Pink1 (1:800), anti-Tom20 (1:10,000), anti-PGC-1α (1:10,000), anti-Podocin (1:600), anti-P62 (1:40,000), anti-Parkin (1:2000), anti-Desmin (1:50,000), anti-SIRT1 (1:1000), and TFAM (1:1000) overnight at 4 °C. The membrane was washed three times with PBST and incubated with the horseradish peroxidase-conjugated secondary antibody for 2 h at room temperature to combine with the primary antibodies. Finally, images of the target protein were developed and collected using a gel imaging system (Bio-Rad, Hercules, CA, USA). Protein bands were visualized by enhanced chemiluminescent (TIANGEN Biotech, Beijing, China) detection reagents. The expressions were quantified by ImageJ software. To eliminate deviations, each assay was repeated at least three times.

### 4.5. RT-PCR

Total RNA was isolated from the cell lines using a TRIzol reagent (TransGen Biotech) and reverse-transcribed into cDNA with an EasyScript^®^ One-Step gDNA Removal and cDNA Synthesis SuperMix (TransGen Biotech) according to the manufacturer’s instructions. RT-PCR was performed using the QuantiNova™ SYBR Green PCR (QIAGEN, Hilden, Germany). All primers were designed and synthesized by Generay Biotech Co., Ltd. (Shanghai, China) and are listed in Table S2. The relative mRNA expression was quantified using the 2^−ΔΔCT^ method. β-actin was used for normalization. Three independent experiments were performed for each sample.

### 4.6. Immunofluorescence

Cells were washed in PBS, fixed with 4% paraformaldehyde for 20 min, made permeable with 0.1% Triton X in PBS for 5 min, and sealed with 3% bovine serum albumin in PBS for 30 min. Subsequently, primary antibody was added overnight at 4 °C, and secondary antibody was added at room temperature for 30 min in darkness. DAPI was incubated for 5 min and then washed with PBS. Finally, we observed the cells under a confocal laser scanning microscope and quantified the results using ImageJ v1.8.0 The average integrated optical density value was used to represent the protein expression.

### 4.7. ΔΨm, ATP Content, and ROS Determination

ΔΨm and ROS were detected using the JC-1 assay kit (Beyotime Biotechnology, Beijing, China) and ROS assay kit (Beyotime Biotechnology), respectively, and then analyzed by flow cytometry. The production of ATP was measured using an ATP assay kit (Nanjing Jiancheng Bioengineering Institute, Nanjing, Jiangsu, China) according to the manufacturer’s instructions.

### 4.8. Transmission Electron Microscopy

Cells were washed in PBS and fixed with 2.5% glutaraldehyde for eight hours. After fixation, cells were rinsed three times with 0.1 M phosphate buffer (pH 7.4) for 15 min each and fixed with 1% osmium acid and 0.1 M phosphate buffer for two hours at room temperature. Then, cells were observed with an electron microscope after dehydration, permeabilization, embedding, sectioning, and staining.

### 4.9. Experimental Animals

Six-week-old male Sprague–Dawley rats (specific pathogen-free grade), 160–180 g, purchased from Hunan SJA Laboratory Animal Co., Ltd. (Changsha, Hunan, China) were injected intraperitoneally with normal saline (control, *n* = 6) or STZ (60 mg/kg body weight). Seventy-two hours after the STZ injection, tail vein blood glucose levels were detected. If the random blood glucose level was higher than 16.7 mmol/L for three consecutive tests, the diabetes model was considered to be successful. Eight weeks after STZ injection, the rats were randomly divided into the following two groups: DKD model group (*n* = 10) and P-MSCs treatment group (tail vein injection, 1 × 10^6^ in 2 mL PBS, per rat, *n* = 10). The animal license number is SYXK (Gan) 2021-0003.

### 4.10. Immunohistochemistry

After dewaxing, rehydration, antigen retrieval, inactivating endogenous peroxidase activity, and blocking, the renal tissue sections were incubated with various primary antibodies: anti-Beclin1 (11306-1-AP, Proteintech), anti-P62 (bs-2951R, Bioss, Woburn, MA, UA), anti-Desmin (ab32362, Abcam), anti-SIRT1 (ab189494, Abcam), anti-PGC-1α (sc-518025, Santa Cruz Biotechnology), and anti-TFAM (AF0531, Affinity Biosciences) at 4 °C overnight. The sections were then incubated with secondary antibody for 30 min after washing with PBS for three times. Diaminobenzidine was used as the chromogen. Finally, sections were stained with hematoxylin and examined using a microscope.

### 4.11. Statistical Analysis

All data collected in our experiments are expressed as means ± standard deviation. Student’s *t* test was used to compare two groups. Three or more groups were compared using one-way analysis of variance. *p* values < 0.05 were considered to be statistically significant.

## 5. Conclusions

This is the first study to investigate whether P-MSCs ameliorate podocyte injury and PINK1/Parkin-mediated mitophagy inhibition, and we found for the first time that P-MSCs play a therapeutic role via the SIRT1-PGC-1α-TFAM signaling pathway. We propose that enhancing PINK1/Parkin-mediated mitophagy and the expression of SIRT1, PGC-1α, and TFAM in podocytes may be a novel strategy for the treatment of DKD.

## Data Availability

All data generated or analyzed during this study are included in this publication.

## Supplementary Materials

The following supporting information can be downloaded at: https://www.mdpi.com/article/10.3390/ijms24054696/s1.

## Abbreviations

DKD	diabetic kidney disease
MSCs	mesenchymal stem cells
P-MSCs	placenta derived mesenchymal stem cells
SIRT1	silencing information regulator 2 related enzyme 1
PINK1	phosphatase and tensin homolog–induced kinase
PGC-1α	peroxisome proliferator-activated receptor γ coactivator-1alpha
TFAM	transcription factor A, mitochondrial
FBS	fetal bovine serum
β-actin	beta actin
HG	high glucose
NC	negative control
siRNAs	small interfering RNAs
PBS	phosphate-buffered saline
PBST	phosphate-buffered saline with Tween 20
WB	Western blot
RT-PCR	reverse transcription–polymerase chain reaction
ΔΨm	mitochondrial membrane potential
ROS	reactive oxygen species
STZ	streptozotocin
