# Supplementary material for: Placental Mesenchymal Stem Cells Alleviate Podocyte Injury in Diabetic Kidney Disease by Modulating Mitophagy via the SIRT1-PGC-1alpha-TFAM Pathway
Source: Int J Mol Sci. 2023 Feb 28;24(5):4696. doi: 10.3390/ijms24054696 (PMC10003373; doi:10.3390/ijms24054696)
Supplement: Supplementary file 1 [file ijms-24-04696-s001.zip › Table S2.pdf]

**Table S2**

Nucleotide sequence of primers for RT-PCR

| Gene name | Forward primer | Reverse primer |
|---|---|---|
| β-actin | CGTTGACATCCGTAAAGACC | AACAGTCCGCCTAGAAGCAC |
| Beclin1 | TGTCTTCAATGCCACCTTC | TTTCATTCCACTCCACAGG |
| LC3B | AGCGTCTCCACACCCATC | GACCAGAACTCCCAGCCA |
| SQSTM1/P62 | GCCAGAGGAACAGATGGA | GGGAGATGTGGGTATAGGG |
| Parkin | TCTTCCAGTGTAACCACCGTC | GGCAGGGAGTAGCCAAGTT |
| PINK1 | ATCAGTAGCATCTAGCATAC | GATCACTGATCAGATCTATCC |
| Tom20 | GCTGCAAGTGTTACAGCAGA | GTCGGAAGCTTGGTCAGAAG |
| Desmin | GAGGGTGTTGGGATCTGC | AGAGAGGGGCCATGAGG |
| SIRT1 | AGAACCACCAAAGCGGAAAA | AATCCCACAGGAGACAGAAACC |
| PGC-1a | CGAGAAGCGGGAGTCTGAAAG | GAGCAGCGAAAGCGTCACA |
| TFAM | CAGGAGGCAAAGGATGATTC | CCAAGACTTCATTTCATTGTCG |
