# Supplementary material for: Placental Mesenchymal Stem Cells Alleviate Podocyte Injury in Diabetic Kidney Disease by Modulating Mitophagy via the SIRT1-PGC-1alpha-TFAM Pathway
Source: Int J Mol Sci. 2023 Feb 28;24(5):4696. doi: 10.3390/ijms24054696 (PMC10003373; doi:10.3390/ijms24054696)
Supplement: Supplementary file 1 [file ijms-24-04696-s001.zip › Figure S1.pdf]

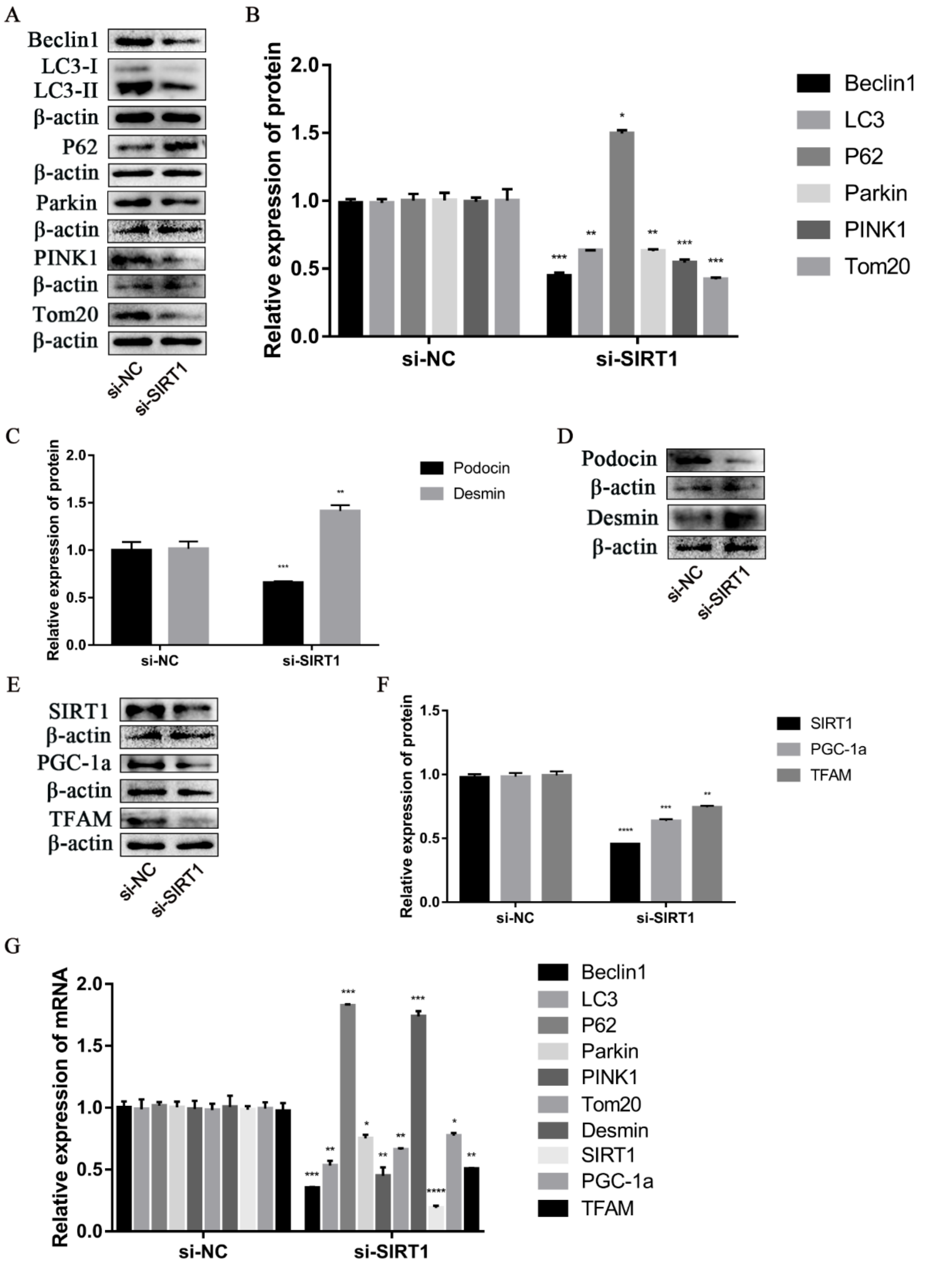

Figure S1. The inhibition of SIRT1 expression aggravated podocyte injury, inhibited PINK1/Parkin-mediated mitophagy, and decreased the expression of PGC-1α and TFAM. A-B: Representative western blots analysis of Beclin1, LC3II/LC3I ratio, P62, Parkin, PINK1 and Tom20 in MPC5. C-D: Representative western blots analysis of Podocin and Desmin in MPC5. E-F: Representative western blots analysis of SIRT1, PGC-1a and TFAM in MPC5. G: Representative RT-PCR analysis of Beclin1, LC3II/LC3I ratio, P62, Parkin, PINK1, Tom20, Desmin as well as

SIRT1, PGC-1a and TFAM in MPC5. β-actin was used as loading control. *$p < 0.05$, **$p < 0.01$, ***$p < 0.001$ and ****$p < 0.0001$ vs. the normal contrast.
